# Technological Aspects of Manufacturing and Control of Gears—Review

**DOI:** 10.3390/ma16237453

**Published:** 2023-11-30

**Authors:** Piotr Boral, Rafał Gołębski, Ruzena Kralikova

**Affiliations:** 1 Faculty of Mechanical Engineering and Computer Science, Department Technology and Automation, Czestochowa University of Technology, Al. Armii Krajowej 21, 42-200 Czestochowa, Poland; piotr.boral@pcz.pl; 2 Faculty of Mechanical Engineering, Kosice University of Technology, Letná 9, 042 00 Kosice, Slovakia; ruzena.kralikova@tuke.sk

**Keywords:** gear machining, tooth profile, tooth line, involute, chiseling, hobbing, planning, grinding, shaving, CNC machine tool, coordinate measuring machine

## Abstract

Gear drives are widely used in various fields and applications due to their properties and capacity. Their versatility, durability, and ability to transmit high torques as well as precision and reliability make them extremely useful in many fields of technology. They are widely used in industrial and energy machinery, vehicle drive systems, aerospace, medical devices, and many other areas. Gears can be manufactured using many technologies. This work focuses mainly on machining with particular emphasis on high-performance new technologies. The process of mathematical modeling of the gear and the machined profile is strongly related to CNC machining technologies. A robust correlation of systems supporting the design and modeling of sliding gears needed for the manufacturing process is presented in the article. It is very important to properly assess gears with correct manufacturing in accordance with a specific standard. The article presents an analysis of available methods for controlling gears using coordinate measurement techniques. Gear machining methods were assessed in terms of the technologies used as well as their productivity and manufacturing tolerance.

## 1. Introduction

Gears are still a very important component of all types of machines and devices used in industries and in everyday life. There are many examples of the use of gears. They are widely used in industrial machines [1,2,3], such as machine tools, presses, paper-making machines, conveyors, electric motors, etc. Gears are an integral part of vehicle drive systems [3,4,5,6]. They can be used in gearboxes of passenger cars, trucks, motorcycles, and construction machines. Gear transmissions allow the rotational speed and torque to be changed, which is necessary for smooth driving under various conditions and loads. In aviation and astronautics, gears are used in various systems, such as propulsion systems of airplanes, helicopters, drones, flight control systems, and landing gear mechanisms [7]. Gears must meet high requirements for strength, precision, and reliability. Gears are also used in various types of medical devices, such as computed tomography scanners, X-ray machines, diagnostic systems, and rehabilitation devices. In these applications, gear drives ensure precise and reliable motion transmission. In the energy industry, both conventional and renewable, gears are used in turbines, generators, water pumps, and other devices. They allow the effective conversion of mechanical energy into electrical or hydraulic energy. In the mining and quarrying industry, spur gears are used in equipment such as mills, crushers, drilling rigs, and belt conveyors. They allow the transfer of heavy loads and the effective grinding, transporting and processing of materials.

During operation, the active gear presses on the passive gear, which causes rotation. The initial tooth of the active gear contacts the top of the tooth of the passive gear, and the contact point later moves along the tooth profile. The cooperation between the active and passive wheels ends when their vertices stop touching each other. The place where the teeth temporarily touch is called the pressure point. The pressure line is formed by subsequent pressure points; on a specific section of this line, the teeth of the active and passive gears cooperate. In order for the gears to work properly, it is necessary to select the appropriate tooth profiles at the design stage. These contours can be of any type, but they must meet the condition of appropriate coupling of the curvatures of the contours of the sides of the teeth of the mating gears. The most frequently used outlines of tooth sides are cycloid and involute [2].

In gear design technology, the following types of tooth profiles can be distinguished:

- Linear: One of the first shapes used on gear wheels, it is currently no longer used in advanced gearing technology. It is found in simple mechanisms that do not require precision of execution.
- Circular: Also one of the first shapes used on gear wheels, it is currently no longer used due to its limited use in the precision of interacting elements.
- Orthocycloids: Formed by a curve drawn by the point of a circle rolling in a straight line, this outline is commonly used in watch mechanisms that do not require precision of workmanship. It has currently replaced the linear and circular outlines.
- Epicycloids: Formed by a curve drawn by a point of a wheel rolling outside another wheel, this outline is also used in watch mechanisms. It replaced the linear and circular outlines but was later replaced by the involute outline.
- Hypocycloids: Formed by a curve drawn by the point of a wheel rolling inside another wheel, this outline is currently used only in watch mechanisms. In the technology of machine gear design, it has been replaced by the involute outline.
- Involute: A curve drawn by the point of a rolling straight line without slipping along another curve, involute is currently the most commonly used outline in gear wheels.
- Circular arc: An involute, a part of which constitutes the lateral outline of the teeth, it is created as a path of any point on a straight line without slipping along the basic circle. This outline is characterized by small pressures between the teeth. In gears used in machine construction, the involute shape is the most common.

**Figure 1:**
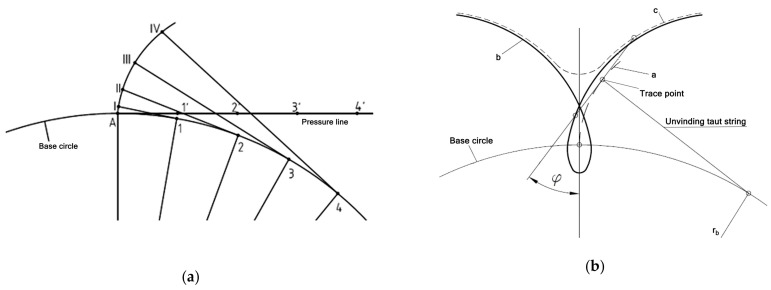
Geometry of an involute wheel: (**a**) constructing an involute; (**b**) involutes: a—ordinary, b—elongated, c—shortened.

In geometry, an involute is any curve traced by a point lying on a straight line that rolls without slipping along any curve. The curve created by a straight line rolling without slipping on the main wheel of a gear wheel is called the involute of the gear or abbreviated to involute (Figure 1). In the meshing theory, curves drawn by points lying in a fixed position outside a straight line rolling in a circle are also considered. On Figure 1, the next points of the involute characteristics started from point A are marked respectively: on base circle 1, 2, 3, 4; pressure line 1′, 2′, 3′, 4′; and, respectively, as points forming the involute curve as I, II, II, IV. They are called extended or shortened involutes depending on the method of formation (Figure 1b). The involute profile meets the basic requirements set for tooth profiles in the theory of mechanisms, i.e., it ensures continuity of movement and stability of the gear ratio, i.e., constancy of the ratios of the instantaneous values of the rotational speeds of both mating wheels [1,2]. Gears with such an outline can be made using the shaped or hobbing method, ensuring not only high efficiency but also high precision. The theory, design, geometry, and production of all types of gears and gear drives are presented by Litvin and Fuentes [1]. Among general topics, the authors present new geometries of modified spur and helical gears, spur gear drives, and new approaches to the design of single-stage planetary gears and helical bevel gears. They present new methods for grinding spur gear drives, generating two-wheel pinions and new types of cylindrical gears.

Radzevich [2] dealt with the design and technology of gears, describing the kinematics of shaping gear wheels with broaches, disc, and finger cutters. He presented methods of generating gear teeth using rotary rack and pinion tools. In [3], Davis presented the materials most often used for gears and methods for improving their mechanical properties. He presented conventional methods of chip and chipless machining of gears.

Materials used to produce gears must meet a number of requirements, such as strength, wear resistance, hardness, and durability. In the case of gear teeth, the surface should be hard with a soft and durable core to provide resistance to wear and fatigue. In recent years, there has been significant progress in the production of steels ideally needed for gears. Gear steels are developed to have completely controlled hardenability, thus reducing distortion and making it precisely predictable and repeatable. Alloy steels are the most preferred material for gears. The choice of the appropriate material depends on the specific application, and the characteristics of the materials may vary depending on the needs. Table 1 shows the characteristics of materials commonly used for the production of gears.

Designing and manufacturing gears poses demanding challenges in maintaining quality at the required level at the lowest possible cost. The goal is to reduce the number of operations or machines through which a workpiece must pass to achieve its final specifications for tooth shape dimensions and quality. Ideally, the process should involve universal means of production and tools, which will also reduce production costs.

## 2. Machining of Gears

Currently, the most common machining of gears is hobbing. During hobbing, the tool generates oscillatory or rotational movements about an axis while the gear being machined is gradually rotated. The tool movements are controlled to create the appropriate tooth profile. Envelope machining allows precise shaping of teeth according to specific profiles and dimensions. Hobbing machining is carried out, among others, for milling with a worm cutter, skiving milling, slotting, planing or grinding, and shaving. Hobbing that is commonly used in conventional technology is based on the principle of rolling motion of the gear wheel on the rack or on the second gear wheel (Table 2).

**Figure 2:**
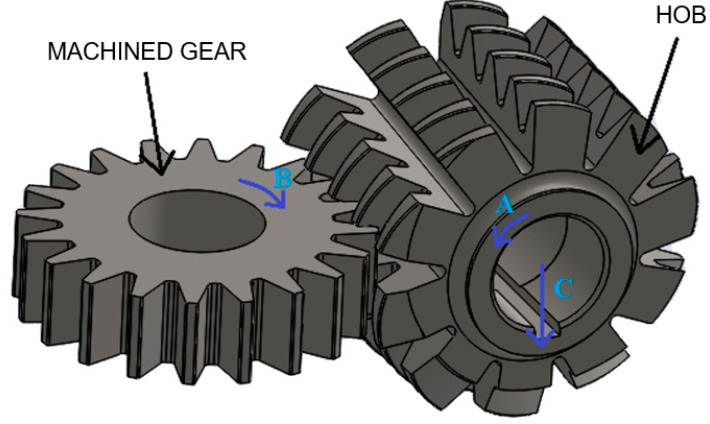
Machining gears by hobbing.

Hobbing milling is performed using a worm cutter, which has a cross-sectional shape similar to a rack, and the cutting edges are arranged along the helical line of the worm cutter. During the rotation of the tool, each subsequent cutting edge of a cutter tooth of the same turn along the cutter axis creates a condition for the feed movement of the rack and pinion tool. The rotation of the screw in direction A is synchronized with the rotation of the workpiece wheel relative to its axis in direction B, and the tool’s movement in direction C goes deeper into the material, shaping the entire wheel (Figure 2). The principle of grinding with a worm-shaped grinding wheel is the same.

**Figure 3:**
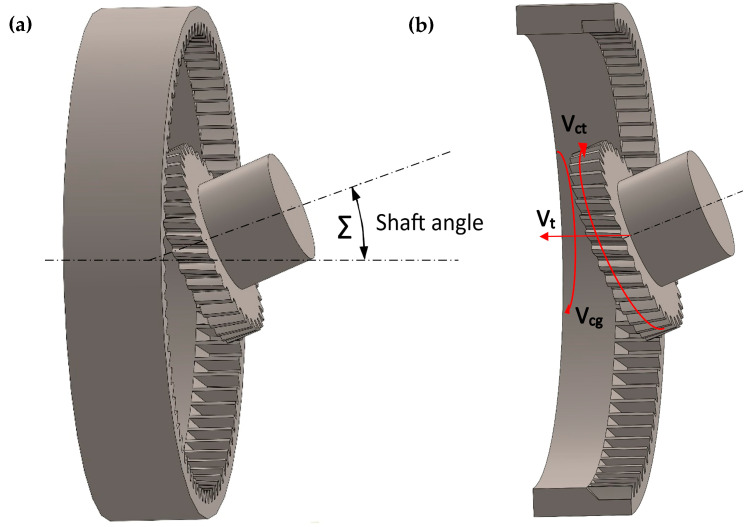
Power skiving machining: (**a**) setting the angle of the tool relative to the axis of the workpiece; (**b**) machining principle.

The power skiving method [8] is a combination of classic hobbing machining and chiseling (Figure 3). The tool in the form of a milling head performs rotational movement, V_ct_, and longitudinal movement, V_t_, while the workpiece rotates around its own axis, V_cg_. Appropriate coupling of two rotational movements of the tool and the workpiece allows the shaping of the tooth contour.

**Figure 4:**
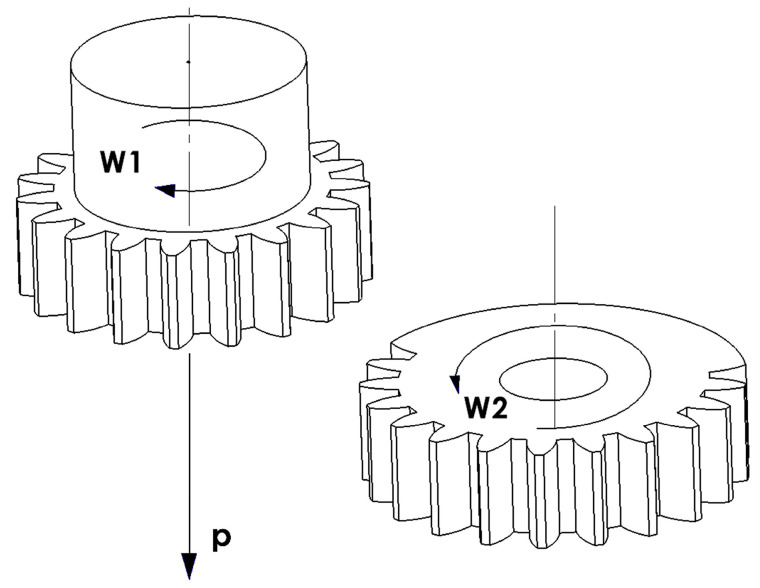
Fellows chiseling: movements of shaping the gear teeth with a modular chisel.

The principle of cutting teeth according to the Fellows method is performed with a tool in the form of a modular chisel [9], which has the shape of a gear wheel with appropriately shaped blade angles. The rolling motion in this method consists of the rotational motion of the tool W_1_ and the rotational motion of the machined wheel W_2_ (Figure 4). In addition to the rolling movements and working movement of the reciprocating tool along the tooth line, there are also radial movements, i.e., a deep feed movement aimed at inserting the tool to the appropriate depth into the material of the cut circle and moving the tool away from the object during the tool’s return stroke.

The key factors that influence the tolerance of the gear wheel are the stiffness and precision of the machine tool, the accuracy of the tools, the relative positioning of the tool and the workpiece during machining, and technological parameters. The machine tool should be stable, free from clearances, and the drive and control systems should operate precisely.

Ding et al. [10] proposed a new method for identifying significant geometric errors of five-axis gear grinders, where the assembly errors of the grinding wheel and gear are integrated into the mapping model. A real digital tooth surface was constructed taking into account geometric errors and the grinding mechanism of the gear shape in accordance with the homogeneous transformation matrix (HTM) and the theory of multibody systems (MBS). The accuracy of the sensitivity analysis results was verified, and the advantages of the proposed improved global method were discussed.

Xia et al. [11] presented a new method for eliminating the gear chain error (TCE) in hobbing machines. Based on error source analysis and discrete Fourier transform (DFT), the measured TCE was first decomposed into various error components, including total harmonic, subtotal harmonic, and others. Then, a classified compensation theory was presented, in which measurement system error compensation (MSEC) and software axis were used to compensate for the first two types of error components, respectively. The frequency response for the C-axis control system was then identified, and the final compensation values were calculated and incorporated into the classified compensation process. The results of the experiment can be the basis for precise hobbing of gears.

Correct shaping and profiling of cutting tools is important to obtain the appropriate tooth profile. Precise profiling of tools allows you to achieve the required shape, inclination, and other geometric parameters. The material from which the gears are made also affects their tolerance manufacturing.

Currently, much attention is paid to mathematical modeling of the cutting and virtual machining processes. The new approach assumes transferring a significant part of the machining analysis to the virtual zone while maintaining a faithful representation of the process. This allows you to identify potential problems that could arise during real machining and thus save related operational costs. Duta et al. [12] presented an experiment on the dynamic behavior of a milling machine during gear hobbing machining. The analysis showed that the displacements were within the permissible limits and did not significantly affect the shape of the tooth profile. Simulation models of the gear hobbing process using the finite element method were developed that would be able to predict the impact of cutting parameters on the gear profile and blade wear. A dynamic simulation of the hoop machining process was carried out in the ANSYS system for a limited period of time, and the values of strain, equivalent strain, and stress were determined. The chip falling off time and the corresponding value of the equivalent stress occurring during chip breakage were determined on the basis of the von Mises theory of maximum strain energy. The FEM method used in the simulation took into account the stresses occurring in the area of contact between the tool and the chip.

Hrytsay et al. [13] described a new method for modeling the process of peripheral shaping of gear teeth. In the proposed method, the algorithm for shaping momentary transition surfaces and computer simulation of 3D chip cutting cross sections were based on the results of the kinematics of hobbing cutting processes and rheological analysis of the hobbing cutting process formation. The obtained 3D geometric models of the cut layers and three dimensional nondeformed chips made it possible to determine the force in two dimensions on the active surface of the cutter’s length, i.e., on its individual turns, and along the angle of attack of the front of the rotation column, i.e., on individual columns. This gives an idea of the load on the cutter in the spatial force field and allows calculation of the cutting force at any point in the cutter’s active space. Corrected cutting forces, which are obtained on the basis of this simulation method, can be used to predict the wear and durability of the worm cutter, analyze the impact of the cutting process on the occurrence of oscillations in the elastic system of the machine tool, and implement process control to ensure the required tolerance of the machined gears.

A simulation of gear hobbing machining and the calculation of the geometry and surface of the workpiece was described by Krömer et al. [14]. The quality of gears is the result of the quality of the tool, the precision of the workpiece, the tool mounting, and the kinematics of the machine. In the study, the dry hobbing process was analyzed, enabling the machining of gears with a quality consistent with the DIN to IT 5 standard. To obtain a more real system, the ideal simulation model was modified by taking into account the tool and mounting tolerances. Based on the given technological parameters, the model simulated the hobbing milling process and calculated the chip thickness and cutting length. In addition, the topography of the gear sides and the geometry at the tooth base as well as the roughness of the transition surface at the tooth base were analyzed and presented. Software can be used to calculate the profile and guide lines of gears. Automatic generation of gear machining programs using CAD/CAM systems can significantly improve and speed up the production process, but it is an advanced process and requires appropriate special program modules. Dedicated programs are often prepared for this purpose or are implemented based on the hardware structure. For example, reconfigurable embedded CNC system platform for gear machining based on the “ARM+ DSP” hardware structure was presented by Han et al. [15]. The automatic parametric programming module was introduced to automatically generate an NC machining program in accordance with the gear parameters, cutting tool parameters, and machining process parameters. The proposed built-in CNC system was experimentally verified on a six-axis hobbing machine.

Different materials have different mechanical and thermal properties, which may affect the precision of machining and the durability and stability of gears in operation.

An analysis of the influence of the radius of the cutting edge and the form factor K of crater wear on the wear of gear hobbing tools made of high-speed steel using the powder metallurgy method (PM-HSS) was presented by Kühn et al. [16]. Based on the research, it was shown that the shape factor K affects the wear, while changing the radius of the cutting edge had no significant effect. Uniform coating thickness can significantly extend tool life. Karpuschewski et al. [17] presented research work on the analysis of thermal load on gears during hobbing gear manufacturing, surface integrity states in various finishing operations of hardened gears, and the possibility of avoiding thermal damage in gear manufacturing through tailored process monitoring and rapid nondestructive analytical techniques.

Zhang et al. [18] conducted an experiment to optimize the grinding parameters of gears made of 20CrMnTiH alloy steel with a grinding wheel with ceramic microcrystalline corundum. The pixel method was used to analyze the grinding principles and examine the grinding parameters that influence the grinding indicators, i.e., grinding wheel speed, grinding wheel frame movement speed, and feed speed. Using the linear multiple regression method, the curves of the influence of grinding parameters on grinding indicators, namely, grinding efficiency, grinding wheel life, and surface roughness, were determined. Ultimately, the multicriteria optimization method was used to comprehensively optimize the grinding process. Comprehensive optimization of grinding parameters improved the precision, efficiency, and economy of the 20CrMnTiH gear grinding process.

Piotrowski et al. [19] presented a geometric analysis based on a universal mathematical model of a worm cutter with replaceable sintered carbide inserts. Based on the developed computational algorithm, an original computer program was created to model the position and geometry of cutting inserts positioned on the rake surface. A generalized case of a folding worm cutter with a flat rake surface not parallel to the cutter axis and rake angles different from zero was analyzed. Methods for correcting the position and geometry of cutting inserts to increase the accuracy of the indexable cutter were presented. Chen et al. [20] presented the construction of a slice cutter tool for machining gears and its mathematical model created on the basis of the theory of spatial geometry and the theory of conjugation of space surfaces. These tests were carried out in order to obtain a slice cutter that meets the requirements for the accuracy of gear meshing. Tools were designed for power skiving machining that avoid interference between the originally designed gear surface and the tool flank or the tool body itself and have a zero or non-negative clearance angle along the entire cutting edge. In this method, the mathematical model of the interference-free tool was derived from the barrel-shaped generating surface. The effectiveness of the proposed method was demonstrated using numerical modeling [21].

To achieve good geometric accuracy, the condition of the surface layer and the smoothness of the tooth surface of the gear is crucial for ensuring proper gear efficiency, minimizing wear, and reducing friction. Additional grinding post-treatment is thus required. Research is being carried out to optimize the grinding parameters of gears and their impact on grinding efficiency, grinding wheel life, and surface roughness [18]. In all cutting processes, the resulting residual stress state on the machined surface is greatly influenced by thermal and dynamic loads. If it is necessary to ensure a special state of compressive residual stress in the gear, additional processes should be used, such as shot blasting or rolling [17].

Currently, the skiving method is used for machining gears, especially those with internal teeth. This method is characterized by high efficiency and relatively high machining accuracy, thanks to which it can significantly shorten the main production times. Due to the complexity of cutting kinematics, tool design must be based on simulation. For this purpose, appropriate programs have been developed so that the full shape of skiving tools can be defined. By simulating the production process using developed programs, you can determine all processing parameters in order to optimally adapt the process. In order to further improve the machining accuracy, a new type of skiving tool with double rake faces has been designed to flexibly meet different requirements for modifying the two sides of the working gear teeth. Mathematical models of the coupled curved surface cutting edges of the skiving tool for turning the sides of the teeth of the work wheel with profile modification have been developed. Algorithms have been created for the cutting tool path with a variable shaft angle in the cutting process in order to eliminate the twisting of the side surfaces of the gear teeth with a modification of the pitch.

The prepared software provides a geometric assessment of the resulting geometry of the workpiece. Spath et al. [22] presented the possibilities of the developed simulation software and the method of manufacturing using the skiving method on the example of cylindrical external gearing. Guo et al. [23] conducted basic research to further improve the machining accuracy. A new type of skiving tool with double rake faces was proposed to flexibly meet different requirements for modifying the two sides of the working gear teeth. A mathematical model of the coupled curved surface cutting edges of a skiving tool for turning the sides of the teeth of the work wheel with a profile modification was developed. A cutting tool path algorithm with a variable shaft angle in the cutting process was proposed to eliminate the rotation of the side surfaces of the gear teeth with a modification of the pitch. Processing simulation was performed to verify the feasibility of the proposed improved skiving methods.

McCloskey et al. [24] presented a new virtual model for predicting the unmachined chip geometry and cutting forces in the power skiving process. The model was validated in cutting tests, where the predicted forces agreed with the measurement results. Additional research has focused on examining elastic deformation and vibration in power skiving and improving the material cutting model to enable accurate process and part quality prediction for roughing and finishing. Bertgs et al. [25] presented a description of the kinematics of gear machining using the skiving method and a simulation algorithm. The description of the kinematics of gear skiving machining included methods for creating simulations of gear skiving machining and defined the coordinate systems necessary to transform the tool profile coordinate system into the workpiece profile coordinate system and vice versa. Based on the construction algorithm, two different gear profiles were simulated: an external spur gear and an internal ring gear. An analytical method for calculating the maximum chip thickness was presented. The maximum chip thickness calculated analytically was compared with the simulated one, showing agreement between the results. The research presented by Tapoglou [26] focused on the development and validation of a CAD-based model for predicting cutting forces in power skiving. The model included all movements of the cutting tool and workpiece and was used to define chip and gear geometries using Boolean operations. The influence of process parameters on the cutting force was examined. It was found that the tool helix angle and the side rake angle had the greatest impact on the direction and magnitude of the cutting force. Minimum tool load was observed at 30° for the side rake and 30° for the helix angle. As expected, the feed rate also played a key role in the magnitude of the cutting forces. Based on a simulation model, the method of creating process maps and selecting optimal cutting conditions was presented. The model results can be used in a number of analytical algorithms, including tooth geometry analysis, chip morphology analysis, and calculation of the cutting force component.

Dedicated power skiving machines are specifically designed to perform power skiving operations. If lower machining quality can be tolerated, power skiving can also be performed on conventional six-axis CNC turning and milling centers. Tsai and Lin [27] presented a simple methodology for automatically generating the NC code required for gear production on a conventional six-axis CNC turning and milling machining center. The main factors determining the linear cutting speed of a power skiving tool were analyzed, and the modified DH notation was used to assess the error characteristics of the machine tool system. The validity of the proposed methodology was demonstrated by machining the internal gear on the six-axis DMG MORI NTX1000 CNC turning and milling center. The work of Bauer and Dix [28] presented an innovative method for machining roof type gears using the power skiving method. For this purpose, the tool and technology were designed using a mathematical process model and then tested. The tests were carried out on the Gildemeister GMX250 linear universal turning and milling center. The process was shown to be able to produce the required geometry with significant improvements in productivity and accuracy.

Tsai et al. [21] developed a new method for designing precise and simple cylindrical power skiving tools. The proposed method provides the possibility of designing a feasible power skiving tool that avoids interference between the originally designed gear surface and the tool flank or the tool body itself and has a zero or non-negative clearance angle along the entire cutting edge. In this method, a mathematical model of the interference-free power skiving tool is derived on the basis of a barrel-shaped generating surface. The effectiveness of the proposed method was demonstrated using numerical modeling. Hrytsay et al. [29] presented the results of a computer simulation of the power skiving cutting process of gears. Based on the kinematics of the process, a method for generating three-dimensional geometric models of undeformed shear layers was developed and their parameters analyzed. The influence of tool geometry and technological parameters on cutting conditions was examined.

Further development of gear hobbing focuses on technology integration, improving the quality, precision, and efficiency of the production process and adapting to growing market expectations in terms of tolerance, durability, and sustainability. Gear hobbing machines are becoming more precise and flexible, enabling more complex tooth shapes. The evolution of tools, including milling cutters and grinding wheels, allows more precise machining, vibration reduction, and improved surface finish. Advances in software enable more complex and precise tooth models and the generation of tool paths with greater accuracy.

The development of power skiving technology is mainly driven by innovations in tools, CAM software, and process monitoring systems, aiming to increase the precision, efficiency, and flexibility of this machining method. New measurement and process control technologies allow even greater precision in tooth processing. Improved CAM algorithms enable the generation of more optimal tool routes. New tools and methods for designing bevel cutters allow greater flexibility in shaping teeth of various shapes and configurations. Improving cooling and lubrication techniques and optimizing cutting parameters allow increased efficiency and durability of tools, which translates into a more efficient production process. The development of CAM systems and automation technologies allows even greater integration of design processes and machine tool programming and monitoring of the production process. Further development of power skiving technology means that this method is used in an increasingly wider range of industries, e.g., in the automotive, aviation, and medical industries.

## 3. New Strategies for Gear Machining

**Figure 5:**
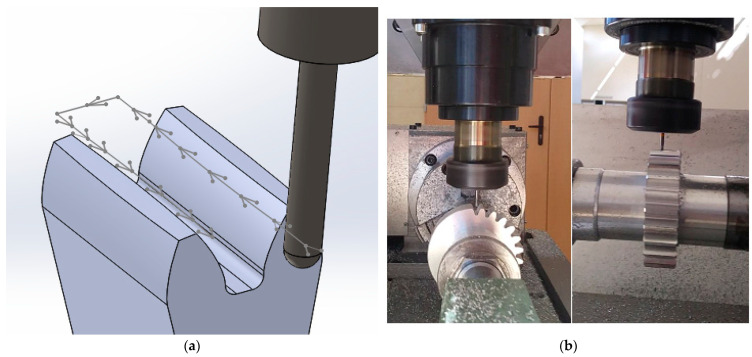
Gear machining using the multipass method: (**a**) tool movement trajectory of one of the paths; (**b**) gear machining.

Conventional strategies used for machining gear teeth rely on specialized machine tools and cutting tools. The quality levels and production times associated with these solutions are generally acceptable, but other elements of the commercial situation, such as production flexibility, are not. They often require specialized tools or equipment that can be expensive and complex to change or modify. If the design requires adjustments to tooth geometry or other types of changes, conventional machining may be limited. The lack of flexibility in converting machines to different types of gears, the long lead time necessary to acquire tools and machines, and the high cost of equipment prevent gear manufacturers from implementing new solutions in terms of quickly responding to customer requirements or switching from the production of one type of gear to another. Tooth machining using the multipass method is performed on a multiaxis CNC machine using a universal tool in the form of an end mill (Figure 5). The lack of geometric dependence of the profile of the processed gear tooth on the contour of the tool allows the production of gears with any contour and shape of tooth line using this method.

Hu et al. [30] presented mathematical models of the developed gear with a curved face and determined the theoretical basis for further research on the machining of gears with a curved face machined using a universal five-axis CNC machining center. However, Bo et al. [31] described a new algorithm on a gear model with curved teeth and verified it using commercial CAD/CAM/CAE software simulations and physical implementations on a universal five-axis CNC machine. Instead of using defined milling tools, they used the shape of the milling tool as a free parameter in their optimization-based approach and, for a given input free form surface (NURBS), developed a custom-shaped tool that performs highly accurate machining. Free-form gear milling is becoming increasingly important as a flexible gear machining process. This is possible thanks to the technological capabilities of multiaxis CNC machines, the large number of degrees of freedom of the tool operation, and the use of universal tool geometry. Staudt et al. [32] presented the characteristics of gears made by free milling and their capabilities compared to conventionally manufactured gears. It has been shown that gears made by free milling provide the same performance as conventional-profile ground gears.

Zhou et al. [33] proposed spur gear technology for a CNC machine based on their software, which directly generates a machining control program from CAD models with given design parameters. A novel envelope approach was introduced, which computed the envelope area of an evolving rectilinear surface as a closed-form (explicit) result. They obtained a representation of the spur gear tooth surface in a closed, rather implicit form. A comprehensive algorithm for generating tool paths was proposed by selecting the largest allowable cutters without the tool cutting into the opposite side of the space between teeth. The algorithm was verified by both simulation and experiment.

Gołębski [34] presented a method of machining a spur gear with straight teeth with an involute profile on a basic DMG MORI CLX350V4 CNC lathe equipped with driven tools. Based on the presented mathematical model, he developed an algorithm for generating a code controlling the machining of cylindrical gears with an involute profile of straight teeth, with the possibility of modifying the transition profile at the tooth base. Likewise, Gołębski et al. [35] presented the multipass method of machining cylindrical gears on universal CNC machine tools. Based on the presented mathematical model, software was developed to generate a code controlling a CNC machine for machining cylindrical gears with a straight and modified tooth line using the multipass method. The tool is a cylindrical end mill with a spherical end, the geometry of which does not depend on the geometry of the cut teeth. The presented profile prismatic model fulfills its function by enabling the prediction and control of cutting strategies and parameters in order to obtain the required contour accuracy. The analyzed technology enables longitudinal modification of teeth. It can be used especially for unit production and machining of wheels with large modules with longitudinal modification of the teeth. Such wheels are insensitive to assembly errors.

Álvarez et al. [36] presented the machining of gears on multitask machines with kinematics of 3 + 2 machining axes using standard tools as a real application of this type of technology due to its flexibility, size, and variety of geometry. General-purpose machines with multitask technology enable machining of the entire gear on one machine by preparing blank parts and cutting gears. The work validated the developed model of surface topography after machining with an end mill with a spherical end surface. The correspondence between the experimentally obtained values and the theoretical values obtained using the model was analyzed.

Malek et al. [37] demonstrated the possibility of precisely manufacturing prototype gears by five-axis milling with universal milling cutters. The impact of tool wear was solved using an alternating milling strategy in which the left and then the right sides were machined first. An innovative tool wear distribution strategy was used with 1, 2, or 4 tools to machine the entire gear wheel. This effectively removed pitch errors by ensuring that sides machined with the new tool would never be close to sides machined with the worn tool. In recent years, many new gear machining strategies have been developed using standard CNC machines and standard cutting tools. Two solutions that have proven particularly effective in enabling machining centers to efficiently cut gears are InvoMilling [38], a gear machining strategy and tool developed by Sandvik, and gearMILL, a software developed by DMG MORI Pfronten [39]. These technologies use the capabilities of multitask machines and CNC machining centers to produce gears with various tooth profiles using one set of tools. The entire workpiece can be processed on one machine without changing the fixture [8].

**Figure 6:**
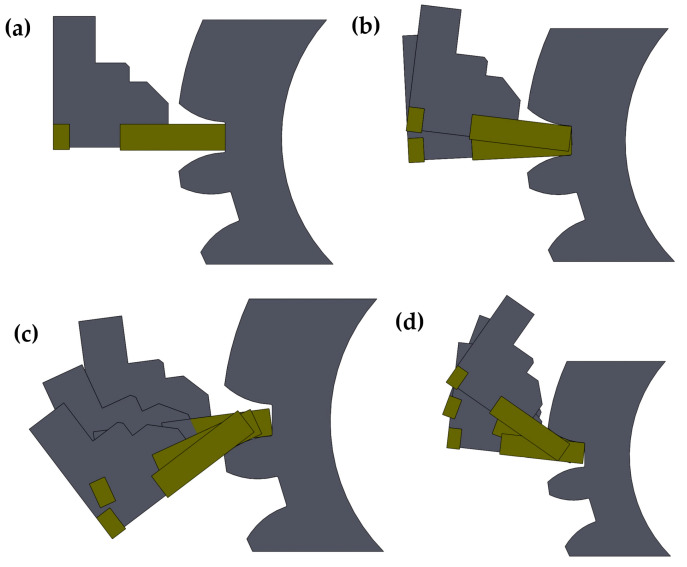
Stages of tooth processing using the InvoMilling method, (**a**,**b**) roughing; (**c**,**d**) finishing.

The processing is carried out in four stages (Figure 6). The first two stages (Figure 6a,b) involve rough machining of the entire area between tooth. The next stage is shaping the tooth base, followed by milling of the lower and upper tooth contours (Figure 6c,d).

**Figure 7:**
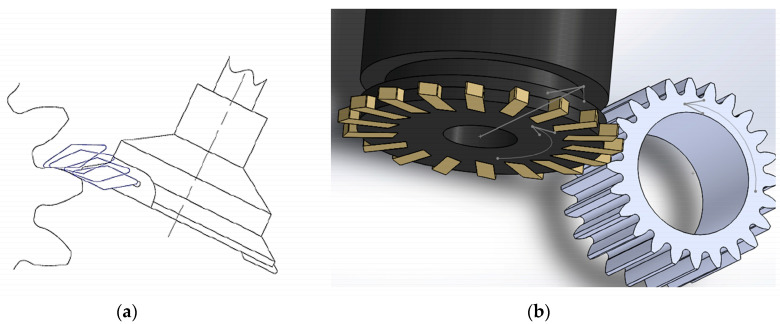
Gear machining using the InvoMilling method: (**a**) tool movement trajectory of one of the paths; (**b**) position of the milling tool during machining [8].

The tool movement trajectory and the position of the milling head when machining the gear teeth are shown in Figure 7.

Hyatt et al. [40] described the InvoMilling and gearMILL machining strategies and compared the manufactured gears in terms of quality and production time with traditional gear manufacturing techniques. The analysis showed that no single gear machining method is best for machining every gear. For example, the length of the machining cycle for gears with small modules is shorter for hobbing and longer for large modules compared to InvoMilling technology. The authors observed that in some cases, multitask machines and CNC machining centers offer even higher efficiency and higher levels of quality. This allows gear manufacturers to optimize their production process.

Scherbarth S. [41] presented a method of milling gears using a cutter with replaceable cutting inserts on the circumference and arranged in such a way that when the cutter is brought to the gear element, they generate gaps between adjacent teeth. The cutting edges of the embedded cutting inserts are positioned radially and perpendicular to the cutter axis. When milling a tooth outline, the cutter axis is set in a plane perpendicular to the contour of a normal tooth (tooth crest), and when inserting cutting inserts, it is rotated around the cutter axis into the surface or gaps between teeth and rotated in this plane in an angular range, including all normal to the profile surface of the manufactured tooth. Glaser et al. [42] presented an investigation on hard finishing of spur gears using the five-axis InvoMilling method developed by Sandvik Coromant. A direct comparison of soft and hard machining was performed on a test gear. The achievable gear and surface properties, spindle moments, and tool wear were compared and assessed. The results of a series of tests showed that with appropriate cutting parameters and cutting materials relative to the inserts, the InvoMilling method is a suitable method for hard finishing of spur gears and very good gear and surface roughness properties can be obtained.

New design solutions for gear transmissions are being developed that cannot be manufactured using conventional machining methods and were previously considered non technological. An example is a bevel gear with a tooth line shaped according to the cosine function [43].

**Figure 8:**
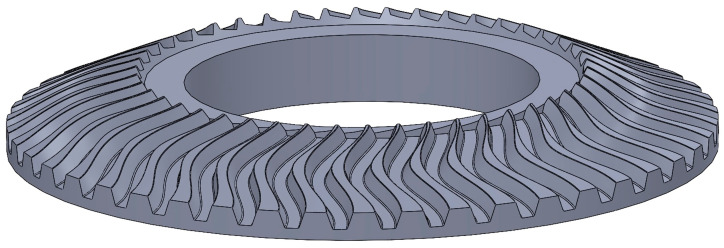
A new solution for the design of the gear profile: model for calculating the profile of bevel gear with a tooth line according to the cosine function.

However, the capabilities of some five-axis CNC milling machines, together with dedicated software for gear machining based on appropriate contour calculations according to mathematical formulas, enable any shaping of not only the tooth line but also the tooth profile (Figure 8).

The cosine tooth profile shown in Figure 8 has a load capacity increased by 40%, which allows the dimensions of the bevel gear to be reduced and thus its weight and moment of inertia. The presented example illustrates the change in perspective on the technological nature of gear design because in the past, the construction of a bevel gear with a tooth line shaped according to the cosine function would have been considered not only not technological but also impossible to produce. Thanks to the technological capabilities of modern machine tools and CAD/CAM software, the designer’s freedom in shaping objects has increased significantly.

## 4. Quality Aspects of Manufactured Gears

In terms of technology, gears belong to the group of geometrically complex products and require high manufacturing precision, which is why a special standard has been developed for them [44]. The ISO 1328-1:2013 [44] and ISO 1328-2:2020 standards [45] establish 12 tolerance classes for gears. For each class, specific requirements are set regarding kinematic accuracy, smoothness of operation, and tooth adhesion. Six types of fits of mating gears in the gear are established, namely, A, B, C, D, E, and H, and eight types of tolerances, i.e., lateral clearances x, y, z, a, b, c, d, and h. The symbols are arranged in the order of decreasing side clearance and its tolerance. There is a relationship between the type of fit and the accuracy class shown in Table 3.

The choice of accuracy class depends on the purpose of the gear wheel and the peripheral speed during gear operation. The kinematic accuracy indicators concern the proportionality of the rotation of the mating gears, while the smoothness of operation and tooth adhesion indicators determine the possibility of vibrations and noise and determine the ability to carry heavy loads. The values of individual indicators are represented, among others, by kinematic deviations of the wheel, radial tooth runout, rotation deviations, measurement inequalities of the wheel axis distance, kinematic deviations of the wheel on the pitch, pitch deviations, tooth contour deviations, and tooth direction deviations. Permissible deviations and tolerance values of the abovementioned indicators are included in the appropriate tables in the ISO 1328-1 and ISO 1328-2 standards depending on the tolerance class.

In order for gears to perform their function satisfactorily, they must be made within certain limits of tolerance corresponding to the intended purpose of the gear. The quality of the gearbox relates to these permissible tolerance limits. Gear quality classes are standardized for various normal module and diameter pitch ranges and other various reference diameter ranges in ISO, AGMA, DIN, JIS, and other standards. AGMA provides eight stages from 15 to 8, where a higher stage number means better gear tolerance. In ISO, DIN, and JIS standards, a lower class number means better gear tolerance [40].

The manufacturing processes used to produce finished gears have certain limitations. The machine, work equipment, cutter, mandrel, machined blanks, and cutting parameters cause certain errors in various gear components. The stages of the production processes must be properly defined. Table 4 provides guidance on the capabilities of various manufacturing processes in terms of achievable quality requirements.

As can be seen in Table 4, InvoMilling and five-axis (gearMILL) methods can produce better quality gears than hobbing-milled ones.

**Figure 9:**
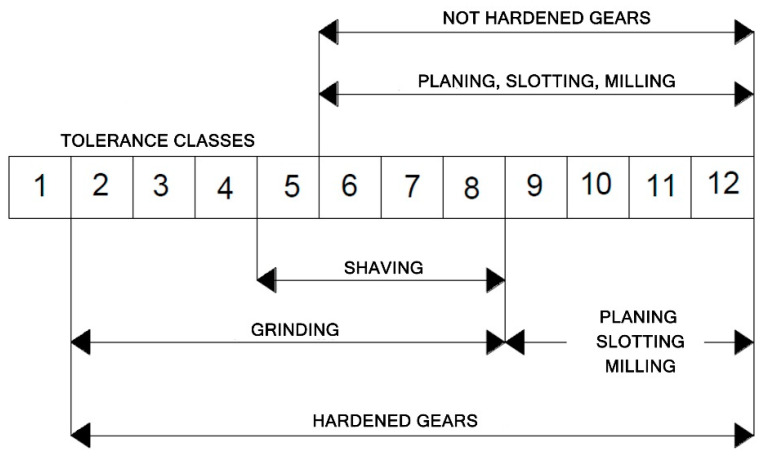
Obtained tolerance classes of gears machined in a soft state and then hardened.

A better tolerance class and lower surface roughness for the manufactured gear can be achieved through finishing. This especially applies to heat-treated gears. Finishing of gear teeth involves machining of gears in a soft condition to a hardness not exceeding 36–40 HRC and subjecting hard gears to heat treatment. The figure below shows what type of machining allows a given tolerance class to be achieved (Figure 9).

Currently, the tolerance of gears is checked on coordinate measuring machines. WPM manufacturers offer a whole range of additional specialized measurement programs for measuring gears. Geometric gear measurement software is crucial in the quality control and gear measurement process. Such software allows accurate measurements of geometric parameters such as size, shape, and properties of teeth. Table 5 shows several popular programs for coordinate geometric measurements with modules for measuring gears.

**Figure 10:**
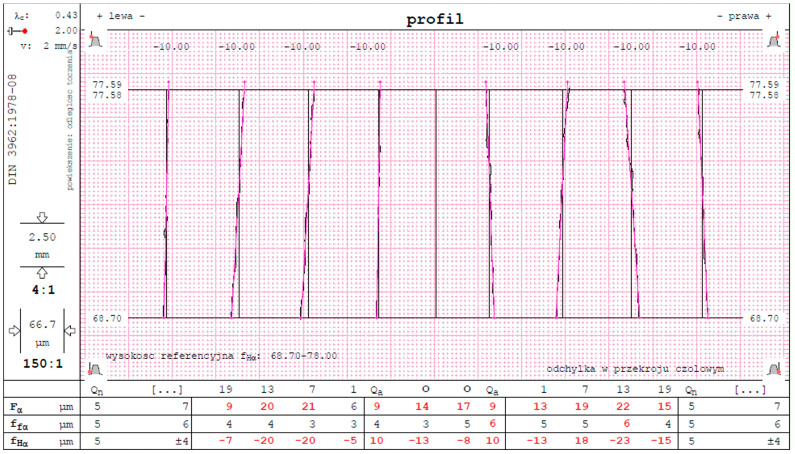
Report from the GearPro involute measurement software.

The accuracy of the methods for checking gears using appropriate software will depend on the design solutions of the measuring devices, i.e., the coordinate measuring machine for which the software has been configured. Before using the measuring machine to measure a gear wheel, it should be calibrated for a given type of measurement and the software being used. The calibration measurement is carried out for a gear wheel made in the “0” tolerance class under the conditions provided for the standard. For example, the GEAR PRO involute program for cylindrical involute gears offered by ZEISS can measure the tooth profile and line, pitch, the diameter of tooth heads, and teeth. It creates an appropriate report and, based on the tolerances, determines the tolerance class of the checked wheel according to a specific parameter [46]. Figure 10 shows a fragment of the report obtained from measuring a gear wheel on a ZEISS coordinate measuring machine using the GEAR PRO involute software (ZEISS, Jena, Germany 2020)

**Figure 11:**
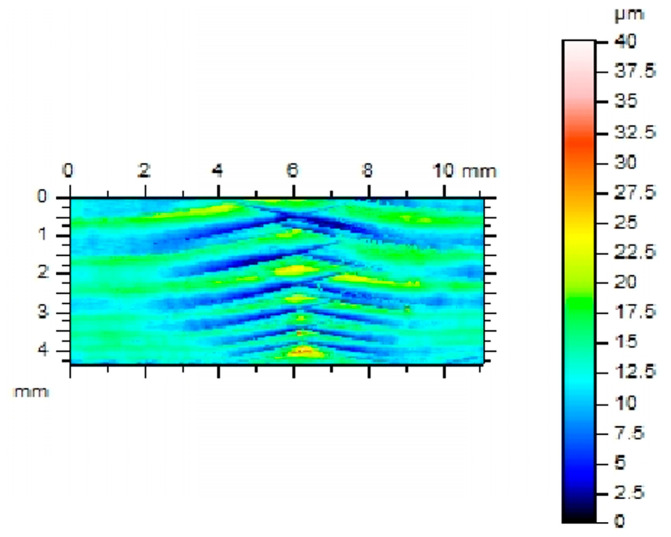
Image measuring the stereometry of the surface after machining using InvoMilling.

**Figure 12:**
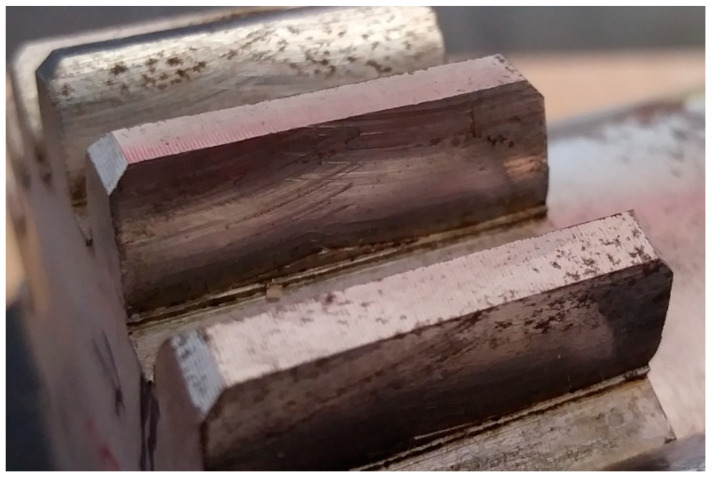
Traces of machining using the InvoMilling method.

In addition to the geometric accuracy of gears, the quality and structure of the side surface of the gear tooth is a very important issue. The geometric structure of the surface of the tooth side of the gear wheels, shaped in the final phase of the technological process, has a significant impact on the surface fatigue strength [47]. An increase in the bearing surface index and the grease retention index contributes to an increase in surface fatigue strength. The most common type of surface wear of mating gear teeth is pitting–chipping. Therefore, parameters characterizing the geometric structure of the tooth side surfaces should be considered. When comparing the geometric structure of the tooth flank surfaces processed using the InvoMilling method (Figure 11), traces of processing are visible even by simple observation (Figure 12).

**Figure 13:**
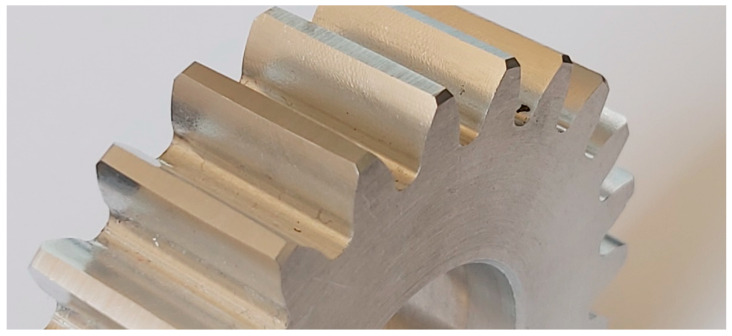
Tool paths after multipass machining of gear with longitudinal tooth modification.

This unevenness is the result of the movement of subsequent cutter blades in the form of arcs when machining the tooth groove. It can be significantly reduced by increasing the number of machining passes, but this increases the machining time. This method can be dedicated to unit or small-batch production of gears. Another new gear machining strategy using universal multitask CNC machines is the multipass method. In this case too, the tolerance of the outline and the smoothness of the machined surfaces depend on the number of machining passes [35]. Traces of subsequent passes of the tool after multipass machining are visible on the side surfaces of the teeth (Figure 13).

**Figure 14:**
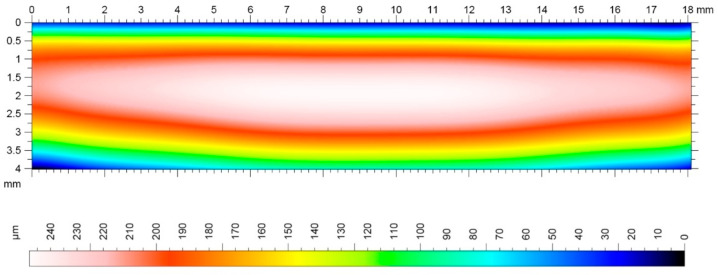
Image of tooth surface stereometry with longitudinal modification machined using the multipass method (barrel tooth).

The advantage of this method over most conventional gear machining methods is that modifications can be made to the tooth contour and line (Figure 14). The tool is a cutter with a geometry independent of the geometry of the machined surface. During machining, the surface and shape of the gear tooth are reproduced by the setting and mutual movement of the tool relative to the machined gear. No special tool is needed for each modification. The possibilities of machining a modified tooth outline are limited only by the kinematic system of the CNC machine tool used.

Gears with straight tooth line are sensitive to misalignment errors of the shafts of the cooperating gears. This problem can be eliminated by its longitudinal modification. For example, by programming the movement of the cutter, you can obtain gears with a slight longitudinal modification, including a version with two convex sides of the tooth (Cv-Cv) and a version with one side of the tooth convex and the other concave (Cv-Cc).

## 5. Discussion

Gear machining is still carried out by specialized companies. The technology is implemented in several stages. In the first stage, all technological procedures are performed from appropriately selected semifinished material to produce a blank part. This process is carried out on universal machine tools using available tools of a typical design. At this point, the tolerance of the machining bases for the implementation of further technological processes is important. Gear machining is carried out as roughing and finishing. During rough machining, gear teeth are cut on the shell using various methods depending on the dimensions and type of gear. The production of gear teeth is carried out on specialized machines that are dedicated only to this purpose using specialized tools. The next stage is finishing, which is carried out immediately after rough machining if the machined wheel is to be in a soft condition or after heat or thermochemical treatment.

The process of machining the entire gear wheel is complicated and requires several specialized and universal machine tools and special tools designed for a specific technology. There is lack of flexibility in terms of quick response to customer requirements. Switching from the production of one type of gear to another requires the manufacturer to incur high costs for the purchase of machines and tools, which also extends the order time.

Currently, the possibility of using multiaxis numerically controlled machine tools using HSC machining and machining strategies enabling machining of hardened materials (e.g., trochoidal machining) allows the manufacturing of a gear wheel on one machine tool. The machining process can be performed on one five-axis milling machine and includes turning, drilling, wheel milling, and rough milling of the tooth in an unhardened state; removing the gear from the machine and heat treating it; and reinstalling the gear on the same machine and finishing it in a hardened state. The possibility of complete machining of finished objects in one technological operation, thanks to multiaxis, multiside machining in one fixing, reduces the cost of production by eliminating expensive tooling and shortening preparatory, setup, and finishing times.

The research presented by Hyatt et al. [40] very accurately compared gear machining methods, hobbing, and InvoMilling in terms of productivity and quality. The analysis showed that no single gear machining method is best for machining every gear. InvoMilling performance also increases as the tooth module increases. For gears with a module of 6 mm and larger, the InvoMilling method is more productive than hobbing. Additionally, in the above analysis, only the cycle time was taken into account. If the total time, which includes setup time and machining time, is taken into account, traditional methods have significant limitations due to longer setup times. In the case of small- and medium-series production, setup time is important. InvoMilling will then be able to obtain better results than hobbing due to the shorter setup time and therefore shorter overall manufacturing time.

Because multitasking machines can apply multiple machining processes and use different tools with automatic changeover, the user has the freedom to choose the machining process based on quality and productivity requirements. The InvoMilling method can be used to produce gears of better quality than hobbing.

Another issue is the low flexibility of conventional technologies due to changes in the design of the details because each modification of the gearing and the use of new design solutions of the gear requires a change of machines and tools. In the most favorable case, when there is even a slight modification of the gear tooth profile, a difficult process of calculation, design, and then production of a new tool that will be used to cut the teeth is required. Therefore, the slightest modification lengthens the manufacturing process and therefore increases manufacturing costs.

Multitasking machines are very flexible. If a parametric or CAD model can be defined for a gear form, the gear can be machined using these machines. Although it cannot be said with certainty, multitask machines offer the customer the best opportunity to make not only today’s types of gears but also those that will be designed in the future. Compared to conventional manufacturing processes, additive manufacturing can significantly reduce energy consumption by using fewer materials and eliminating several steps. But in order to improve the surface quality after sintering, gears nowadays need to be postprocessed. The development of this technology towards more controlled powder sintering and the possibility of obtaining an appropriate quality of surface layer may make this technology a future option for the production of gears.

Continuous research and development of new technologies and materials have allowed the use of additive methods in the production of elements with complex shapes [48]. Additive methods allow the creation of light and strong structures, which is particularly important in the aviation and automotive industries. Also, for the production of prototype gears with complex shapes, 3D printing technologies are increasingly being used. One of the most important methods that is increasingly being used is selective laser melting, a process of melting metal powder as a raw material that is deposited in a layer-by-layer manner. [49]. A study conducted by Tezel et al. [50] compared the operational properties of steel gears manufactured using additive and conventional methods. The gears were subjected to identical testing processes and compared with each other. The density and hardness of gears manufactured using additive manufacturing (direct metal laser sintering—DMLS) were quite similar to those manufactured using conventional methods (hobbing). As a result, gears produced by additive manufacturing were found to wear more.

Recently, we have noticed a change in the approach of many parts manufacturers regarding their reuse and thus possible repair. Quite recently, repairing worn-out machine elements was simply unprofitable. SIEMENS and CHIRON [51] have completed research on the feasibility of using additive manufacturing for cost-effective gear renovation. Laser metal deposition (LMD) technology was used here. The operation allows you to automatically change the material deposition head during the process to meet various workpiece requirements, such as surface quality, control of deposition rate, or material coating. The process may use wire or powder as filler material at various stages of production. The technology increases machining efficiency by up to 30% combined with advanced additive and subtractive capabilities. The comprehensive technology enables machine manufacturers to offer technology for the rapid and flexible refurbishment of worn parts at a competitive price.

## 6. Conclusions

There is no doubt that gears are among the most demanding in terms of technological design and production of machine and device parts.

Also, progress in the field of machine control systems and close integration of gear design technology with the possibility of its implementation on a numerically controlled machine tool is making the use of computer-aided manufacturing techniques in gear manufacturing processes more and more popular.

Based on the content of the analysis conducted in the field of technological aspects of gear manufacturing and control, a number of comparative conclusions can be presented regarding past and current trends in development in the following areas.

In the area of gear design and construction:

- The basics of gear geometry construction and tooth profiles have practically not changed. There is a noticeable tendency in gear design to move away from standardized solutions.
- The involute shape of the gear is still one of the most frequently constructed designs. On this basis, a number of modifications are made, both to the tooth line and its outline.
- Thanks to the extensive use of engineering calculation methods and the possibilities of the TCA (tooth contact analysis) procedure, the gear design process is closely integrated with its production process.
- In engineering tasks, significant progress is noticeable due to the increasingly widespread use of computer computational techniques and thus the development of mathematical models describing more complex kinematic systems of machine tools for machining gears with any geometric characteristics.

In the area of gear manufacturing:

- The technological basis of gear production has not changed, with machining still playing a major role in the process.
- The construction of cutting tools and their geometric aspects have been significantly developed. For gear machining, in addition to special tools such as modular hobs, thanks to the development of CNC machining areas, universal tools made of modern materials are widely used for machining gears. Tools with free geometry that are not geometrically related to the shape of the processed contours are also increasingly being used.
- The common idea for machining gears is to use tool machines with an internal kinematic chain based on gears, allowing machining by creating technological cooperation between the machining tool and the workpiece. Due to the use of numerically controlled machine tools, such a solution is currently not applicable. Commonly used methods include solutions that support computer-assisted preparation of gear machining based on the mathematical generation of processed paths consistent with the designed shape of the machined outline.
- The use of CNC machine tools in the production of gears is related not only to the development of technological machines but also to significant changes in approach in the areas of modern production organization with the simultaneous expansion of production capacity.

In the area of gear control:

- The quality standards for gear manufacturing have not changed. There are 12 defined tolerance classes for gears, while 6 types of fits of mating gears in the transmission have been determined. The relevant standards specify permissible tolerances depending on the gear modules and their sizes.
- In metrological testing of gears, coordinate measuring techniques using modern coordinate measuring machines with dedicated software play a leading role. This solution allows very accurate checking of many parameters of the tested gear while maintaining the measurement in accordance with the desired standard.

## Figures and Tables

**Table 1:** Characteristics of materials used for the production of gears.

Steel 40Cr Steel 20CrMnTi Steel 17CrNiMo6	Characterized by high strength, hardness, and wear resistance. Steel is also relatively easy to work with.
Stainless steel	For applications requiring corrosion resistance, such as in the food and pharmaceutical industries.
Acid-resistant steels	Used in applications where aggressive chemical environments occur.
Specialized alloy steels CrMoV	Used in applications where resistance to high temperatures and high dynamic loads is required.
Gray cast iron and ductile cast iron	Characterized by a better ability to dampen vibrations than steel but are less durable than steel.
Aluminum alloys	Lighter than steel but less durable and usually not suitable for heavy-duty applications.
Polymer plastics	Used in applications that require quiet operation. They are characterized by good lubrication but are much less durable than metals.
Ceramic materials	Used where exceptional hardness and wear resistance are required.
Composite materials	Composite materials that combine different materials, such as carbon fibers in epoxy resins, are used in applications where weight and strength are key factors.
Powder materials	Metallic powders and other powder materials are used in additive manufacturing technologies for gears.

**Table 2:** Classification of gear removal methods using the hobbing method.

Machining Method	Tool	Machine Tool	Application
Planing Slotting Grinding	A cutting tool or grinding wheel in the shape of a single tooth of a rack	Special planer or grinder	Cylindrical and conical gears with straight and helical teeth
Planing Slotting	Rack cutting tool	Special planer Special vertical slotter	Cylindrical and bevel gears with straight and helical teeth
Slotting	Pinion cutter—a tool in the shape of a gear tooth outline	Fellows slotter	Cylindrical and conical gears with straight and helical teeth and external and internal teeth
Milling Grinding	Cylindrical modular hob grinding wheel with outline consistent with the machined profile	Milling and grinding hobbing machine	Cylindrical gears with straight and helical teeth

**Table 3:** The relationship between the type of fit and the accuracy gear class.

Type of Fit	A	B	C	D	E	H
Accuracy class	3–12	3–11	3–9	3–8	3–7	3–7

**Table 4:** Tolerance classes of gears machined using specific production methods.

ISO/DIN Tolerance Class	Grinding	Hard Machining	CNC 5 Axis Machining	Shaving Machining	Shaping/Rack Tool	Shaping/Disc Tool	InvoMilling	Hobbing—Hob Class AA	Hobbing—Hob Class A	Hobbing—Hob Class B	CBN Form Finish
0/1	x										
2											
3		x	x				x				x
4				x	x			x			
5						x			x		
6										x	
7											
8											

**Table 5:** Programs for checking the geometric features of gears.

Software	Manufacturer
Zeiss CALYPSO	ZEISS (Jena, Germany)
MarWin	Mahr GmbH (Goettingen, Germany)
Gear Design Pro	Dontyne Systems Ltd. (Newcastle upon Tyne, UK)
PC-DMIS Gear	Hexagon (Stockholm, Sweden)
Software for Klingelnberg Precision Measuring Centers	Klingelnberg (Zürich, Switzerland)

## Data Availability

Data are contained within the article.

## References

[1] Litvin F.L., Fuentes A. (2004). Gear Geometry and Applied Theory.

[2] Radzevich S.P. (2010). Gear Cutting Tools, Fundamentals of Design and Computation.

[3] Davis J.R. (2005). Gear Materials, Properties, and Manufacture.

[4] Chen Y. (2021). Automotive Transmissions: Design, Theory and Applications.

[5] Naunheimer H., Bertsche B., Ryborz J., Novak W. (2011). Automotive Transmissions.

[6] Lachner G., Naunheimer H. (1999). Automotive Transmissions Fundamentals, Selection, Design and Application.

[7] Smith K.L. (2015). *Aircraft Propulsion*–*Second edition*S. Farokhi John Wiley and Sons, The Atrium, Southern Gate, Chichester, West Sussex, PO19 8SQ, UK. 2014. 1011pp. Illustrated. £63.95. ISBN 978-1-118-80677-7. Aeronaut. J..

[8] Sandvik. https://www.sandvik.coromant.com/pl-pl/knowledge/milling/gear-manufacturing.

[9] Wokar. https://wokar.bls.pl/ostrzenie-tlutakow.html.

[10] Ding S., Chen Z., Zhang H., Yang W., Wu W., Song A. (2023). Gear evaluation deviations-based crucial geometric error identification of five-axis CNC gear form grinding process. J. Manuf. Process..

[11] Xia C., Wang S., Long T., Ma C., Wang S. (2020). Transmission chain error elimination for gear hobbing machines based on classified compensation theory and frequency response identification. Measurement.

[12] Duta A., Ghionea I.D., Popa D.L., Soos L. (2022). Influence of Contact Surfaces’ Impact on the Gear Profile during Hobbing Process. Appl. Sci..

[13] Hrytsay I., Stupnytskyy V., Topchii V. (2019). Improved Method of Gear Hobbing Computer Aided Simulation. Arch. Mech. Eng..

[14] Krömer M., Sari D., Löpenhaus C., Brecher C. (2017). Surface Characteristics of Hobbed Gears. Gear Technology.

[15] Han J., Wu L., Yuan B., Tian X., Xia L. (2017). A novel gear machining CNC design and experimental research. Int. J. Adv. Manuf. Technol..

[16] Kühn F., Hendricks S., Troß N., Brimmers J., Bergs T. (2023). Experimental analysis on the influence of the tool micro geometry on the wear behavior in gear hobbing. Int. J. Adv. Manuf. Technol..

[17] Karpuschewski B., Beutner M., Eckebrecht J., Heinzel J., Hüsemann T. (2020). Surface integrity aspects in gear manufacturing. Procedia CIRP.

[18] Zhang S., Zhang G., Ran Y., Wang Z., Wang W. (2019). Multi-Objective Optimization for Grinding Parameters of 20CrMnTiH Gear with Ceramic Microcrystalline Corundum. Materials.

[19] Piotrowski A., Gołebski R., Boral P. (2020). Geometric Analysis of Composite Hobs. Trans. FAMENA.

[20] Chen X.C., Li J., Lou B.C. (2013). A study on the design of error-free spur slice cutter. Int. J. Adv. Manuf. Technol..

[21] Tsai C.-Y. (2021). Power-skiving tool design method for interference-free involute internal gear cutting. Mech. Mach. Theory.

[22] Spath D., Hühsam A. (2022). Skiving for high-performance machining of periodic structures. CIRP Ann..

[23] Guo Z., Xie R., Guo W., Han W., Gao F., Zhang Y. (2023). A Novel Method for Improving the Skiving Accuracy of Gears with Profile and Lead Modifications. Machines.

[24] McCloskey P., Katz A., Berglind L., Erkorkmaz K., Ozturk E., Ismail F. (2019). Chip geometry and cutting forces in gear power skiving. CIRP Ann..

[25] Bergs T., Georgoussis A., Löpenhaus C. (2020). Development of a numerical simulation method for gear skiving. Procedia CIRP.

[26] Tapoglou N. (2021). Development of Cutting Force Model and Process Maps for Power Skiving Using CAD-Based Modelling. Machines.

[27] Tsai C.Y., Lin P.D. (2018). Gear manufacturing using power-skiving method on six-axis CNC turn-mill machining center. Int. J. Adv. Manuf. Technol..

[28] Bauer R., Dix M. (2022). Novel method for manufacturing herringbone gears by power skiving. Procedia CIRP.

[29] Hrytsay I., Stupnytskyy V., Slipchuk A. (2023). Simulation of a Power Skiving Gear Cutting Process. Strojnícky časopis. J. Mech. Eng..

[30] Hu Y., Lin C., Li S., Yu Y., He C., Cai Z. (2021). The Mathematical Model of Curve-Face Gear and Time-Varying Meshing Characteristics of Compound Transmission. Appl. Sci..

[31] Bo P., González H., Calleja A., López de Lacalle L.N., Bartoň M. (2020). 5-axis double-flank CNC machining of spiral bevel gears via custom-shaped milling tools—Part I: Modeling and simulation. Precis. Eng..

[32] Staudt J., Löpenhaus C., Klocke F. (2017). Performance of Gears Manufactured by 5-Axis Milling. Gear Technology.

[33] Zhou Y., Wang S., Wang L., Tang J., Chen Z.C. (2019). CNC milling of face gears with a novel geometric analysis. Mech. Mach. Theory.

[34] Gołębski R. (2022). Experimental Method of Machining Gears with an Involute Profile Using CNC Lathe with Driven Tools. Materials.

[35] Gołębski R., Boral P. (2021). Study of Machining of Gears with Regular and Modified Outline Using CNC Machine Tools. Materials.

[36] Álvarez Á., Calleja A., Arizmendi M., González H., Lopez de Lacalle L.N. (2018). Spiral Bevel Gears Face Roughness Prediction Produced by CNC End Milling Centers. Materials.

[37] Malek O., Mielnik K., Martens K., Jacobs T., Bouquet J., Auwers W., Ten Haaf P., Lauwers B. (2016). Lead Time Reduction by High Precision 5-axis Milling of a Prototype Gear. Procedia CIRP.

[38] Sandvik Coromant. https://www.sandvik.coromant.com/en-gb/tools/digital-machining/coroplus-tool-path/invomilling/.

[39] DMG MORI Technology Cycles. https://pl.dmgmori.com/resource/blob/44740/36940141e5309142bddc955aae5de4cc/ps0uk-dmg-mori-technology-cycles-pdf-data.pdf.

[40] Hyatt G., Piber M., Chaphalkar N., Kleinhenz O., Mori M. (2014). A Review of New Strategies for Gear Production. Procedia CIRP.

[41] Scherbarth S. (2016). Tooth Milling Cutter and Method for Milling the Teeth of Toothed Gear Elements. U.S. Patent.

[42] Glaser T., Körner T., Sapparth J., Wennmo M. (2018). Hard Machining of Spur Gears with the InvoMillingTM Method. J. Manuf. Mater. Process..

[43] Honczarenko J. (2011). Modern machine tools a technological efficiency of the structure. Mechanik.

[44] (2013). Cylindrical Gears. ISO System of Flank Tolerance Classification. Part 1: Definitions and Allowable Values of Deviations Relevant to Flanks of Gear Teeth.

[45] (2020). Cylindrical Gears. ISO: System of Flank Tolerance Classification. Part 2: Definitions and Allowable Values of Double Flank Radial Composite Deviations.

[46] ZEISS Industrial Metrology. https://www.zeiss.pl/metrologia/produkty/software/calypso-overview/gear-pro.html.

[47] Zwolak J. (2009). Geometric structure of the gear teeth side surface and the process of fatigue wear. Tribilogia.

[48] Fidan I., Huseynov O., Ali M.A., Alkunte S., Rajeshirke M., Gupta A., Hasanov S., Tantawi K., Yasa E., Yilmaz O. (2023). Recent Inventions in Additive Manufacturing: Holistic Review. Inventions.

[49] Svetlizky D., Das M., Zheng B., Vyatskikh A.L., Bose S., Bandyopadhyay A., Schoenung J.M., Lavernia E.J., Eliaz N. (2021). Directed energy deposition (DED) additive manufacturing: Physical characteristics, defects, challenges and applications. Mater. Today.

[50] Tezel T., Topal E.S., Kovan V. (2020). Failure analysis of 3D-printed steel gears. Eng. Fail. Anal..

[51] SIEMENS. https://www.siemens.com/pl/pl/o-firmie/case-study/odnawianie-zamiast-wymiany.html.

